# Individual and Combined Effect of Zearalenone Derivates and Beauvericin Mycotoxins on SH-SY5Y Cells

**DOI:** 10.3390/toxins12040212

**Published:** 2020-03-27

**Authors:** Fojan Agahi, Guillermina Font, Cristina Juan, Ana Juan-García

**Affiliations:** Laboratory of Food Chemistry and Toxicology, Faculty of Pharmacy, University of Valencia, Av. Vicent Andrés Estellés s/n, 46100 Burjassot, València, Spain; agahifozhan@gmail.com (F.A.); crisjua3@uv.es (G.F.); ana.juan@uv.es (A.J.-G.)

**Keywords:** SH-SY5Y cells, zearalenone derivates, beauvericin, MTT, qTOF–MS/MS

## Abstract

Beauvericin (BEA) and zearalenone derivatives, α-zearalenol (α-ZEL), and β-zearalenol (β-ZEL), are produced by several *Fusarium* species. Considering the impact of various mycotoxins on human’s health, this study determined and evaluated the cytotoxic effect of individual, binary, and tertiary mycotoxin treatments consisting of α-ZEL, β-ZEL, and BEA at different concentrations over 24, 48, and 72 h on SH-SY5Y neuronal cells, by using the MTT assay (3-(4,5-dimethylthiazol-2-yl)-2,5diphenyltetrazoliumbromide). Subsequently, the isobologram method was applied to elucidate if the mixtures produced synergism, antagonism, or additive effects. Ultimately, we determined the amount of mycotoxin recovered from the media after treatment using liquid chromatography coupled with electrospray ionization–quadrupole time-of-flight mass spectrometry (LC–ESI–qTOF-MS). The IC_50_ values detected at all assayed times ranged from 95 to 0.2 μM for the individual treatments. The result indicated that β-ZEL was the most cytotoxic mycotoxin when tested individually. The major effect detected for all combinations assayed was synergism. Among the combinations assayed, α-ZEL + β-ZEL + BEA and α-ZEL + BEA presented the highest cytotoxic potential with respect to the IC value. In individual treatment, α-ZEL was the most recovered mycotoxin; while, this was observed for BEA in binary combination α-ZEL + BEA.

## 1. Introduction

Mycotoxins represent one of the most important categories of biologically produced natural toxins with potential effects on human and animal health. The worldwide contamination by these natural products of food, feed, and environment, represents a health risk for animals and humans [1].

Several *Fusarium* species produce toxic substances of considerable concern to livestock and poultry producers. The mycotoxins beauvericin (BEA) and zearalenone (ZEN) and their derivatives (α-zearalenol (α-ZEL), β-zearalenol (β-ZEL), zeranol, taleranol, and zearalanone) can be produced by several *Fusarium* species (mainly *Fusarium graminearum*, but also *Fusarium culmorum*, *Fusarium cerealis*, *Fusarium equiseti*, and *Fusarium semitectum*) that grow on crops in temperate and warm-climate zones [2]. These fungi are present in almost all continents, can grow under poor storage conditions, and mainly contaminate cereal grains, such as maize, wheat, oats, soybeans, and their derived food products [3,4]. 

It has been proved that ZEN and α-ZEL bind to human estrogen receptors and elicit permanent reproductive tract alterations, and consequently, chronical exposure to ZEN present contaminated food can be a cause of female reproductive changes as a result of its powerful estrogenic activity [5,6,7,8]. It has been also reported that ZEN induces genotoxic effects by induction of DNA adducts, DNA fragmentation, and apoptosis [9,10]. As reported by Dong et al. (2010) [5], metabolic conversion of ZEN mycotoxin to α-ZEL and β-ZEL was found in almost all tissues and occurred more efficiently to α-ZEL than to β-ZEL; these mycotoxins are endocrine disruptors which affect steroid hormones such as progesterone [7]. In 2016, EFSA (European food Safety Authorities) indicated that there is a high uncertainty associated with the exposure to ZEN and its modified forms and so that it would rather overestimate than underestimate any risk associated with exposure to modified ZEN [8]. Also, recent studies have indicated that ZEN is immunotoxic [4,11,12] and cytotoxic in various cell lines by inhibiting cell proliferation and increasing ROS (reactive oxygen species) generation [13,14,15].

On the other hand, BEA causes cytotoxic effects by reducing cell proliferation in a time- and concentration-dependent manner [16,17]. Moreover, it can increase ROS generation and lipid peroxidation and produces oxidative stress and depletion of antioxidant cellular mechanisms [14,18,19]. 

Neurotoxicological testing is mainly based on experimental animal models, but several cell lines and tissue culture models have been developed to study the mechanism of neurotoxicity. In general, cells of human origin are attractive alternatives to animal models for the exploration of toxicity to humans. Nonetheless, there are few studies about the effect of mycotoxins at the neuronal level [6,20,21,22].

Regarding the important role of the food industry in human health, studying the impact of mycotoxins and their combinations in feed and food commodities has gained attention over the last few years, due to the ability of most *Fusarium* spp. to simultaneously produce different mycotoxins [23,24,25]. Hence, EFSA has recently published a draft guidance document where a harmonized risk assessment methodology for combined exposure to multiple chemicals in all relevant areas is described [26].

Due to the importance of dietetic exposure to various mycotoxins and their impacts on human’s health, there is an increasing concern about the hazard of co-occurrence of mycotoxins produced by *Fusarium* and of co-exposure to them through diet. Many studies have been conducted on the toxicity of individual mycotoxins; however, few studies have been dedicated to the toxicological interaction of mycotoxins when present in double and triple combinations on different cell lines [16,17,18,27,28,29].

The objective of the present study was to investigate the cytotoxicological interactions between α-ZEL, β-ZEL, and BEA mycotoxins in human neuroblastoma SH-SY5Y cells, via the MTT assay. The effects of combinations of two and three mycotoxins were evaluated by isobologram analysis [30] to determine whether their interaction was synergistic, additive, or antagonistic, as well as to understand how mycotoxins can act at the cellular level.

## 2. Results

### 2.1. Cytotoxicity Assay of Individual and Combined Mycotoxins 

**Figure 1:**
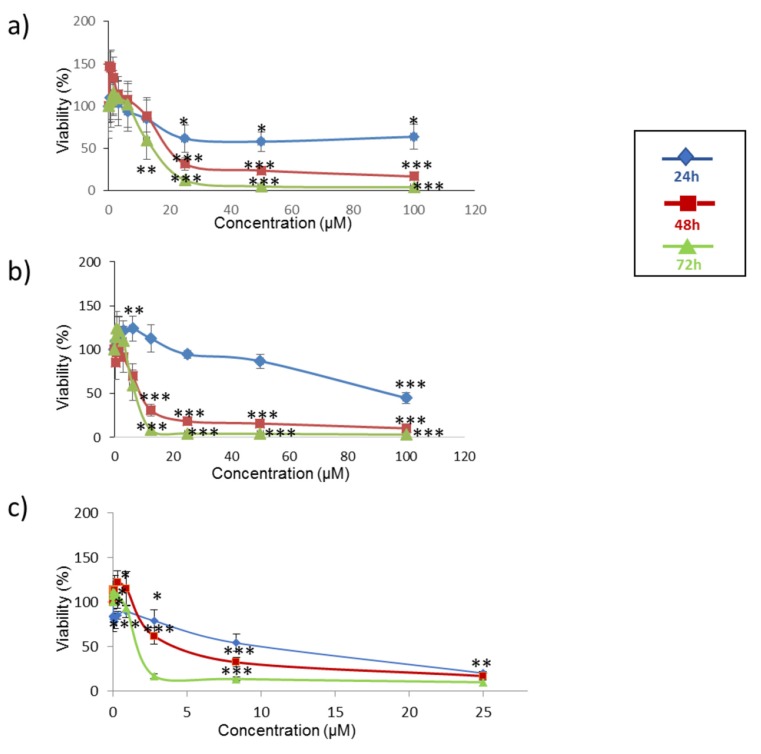
Cytotoxicity of the mycotoxins α-ZEL (**a**), β-ZEL (**b**), and BEA (**c**) individually at 24 h, 48 h, and 72 h. All values are the results of three independent experiments with eight replicates and are expressed as mean ± SD; *p* ≤ 0.05 (*), *p* ≤ 0.01 (**), *p* ≤ 0.001 (***).

The cytotoxicity effects of α-ZEL, β-ZEL, and BEA mycotoxins on SH-SY5Y cells were evaluated by the MTT assays over 24, 48, and 72 h. Figure 1 shows the time- and concentration-dependent decrease in cell viability after exposure to each mycotoxin individually, while IC_50_ values are shown in Table 1. After 24 h, the IC_50_ value could be calculated only for β-ZEL and was 94.3 ± 2.0 µM; after 48 h of exposure, the IC_50_ values were 20.8 ± 0.5 µM for α-ZEL and 9.1 ± 1.8 µM for β-ZEL. After 72 h of exposure, the IC_50_ values were 14.0 ± 1.8 µM, 7.5 ± 1.2 µM. and 2.5 ± 0.2 µM for α-ZEL, β-ZEL, and BEA, respectively. According to the IC_50_ values obtained at 72 h, BEA showed the highest cytotoxic effect on SH-S5Y5 cells (Table 1).

**Figure 2:**
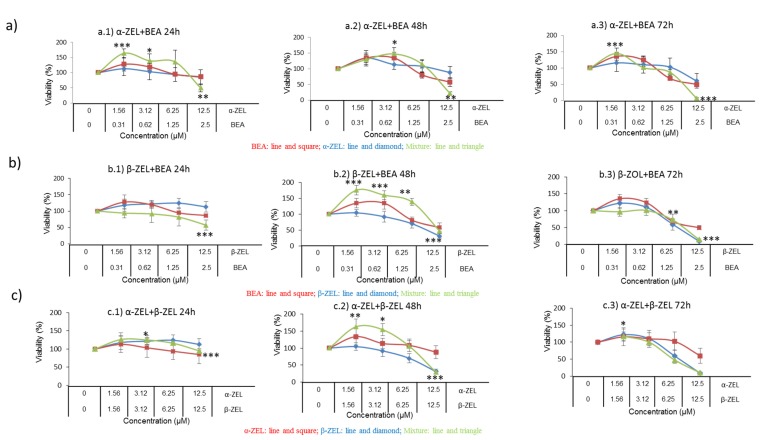
Cytotoxicity of the mycotoxin combinations of α-ZEL + BEA (5:1) (**a**), β-ZEL + BEA (5:1) (**b**), and α-ZEL + β-ZEL (1:1) (**c**) at 24 h (a.1, b.1, and c.1), 48 h (a.2, b.2, and c.2) and 72 h (a.3, b.3, and c.3). All values are the results of three independent experiments with eight replicates and are expressed as mean ± SD; *p* ≤ 0.05 (*), *p* ≤ 0.01 (**), *p* ≤ 0.001 (***).

**Figure 3:**
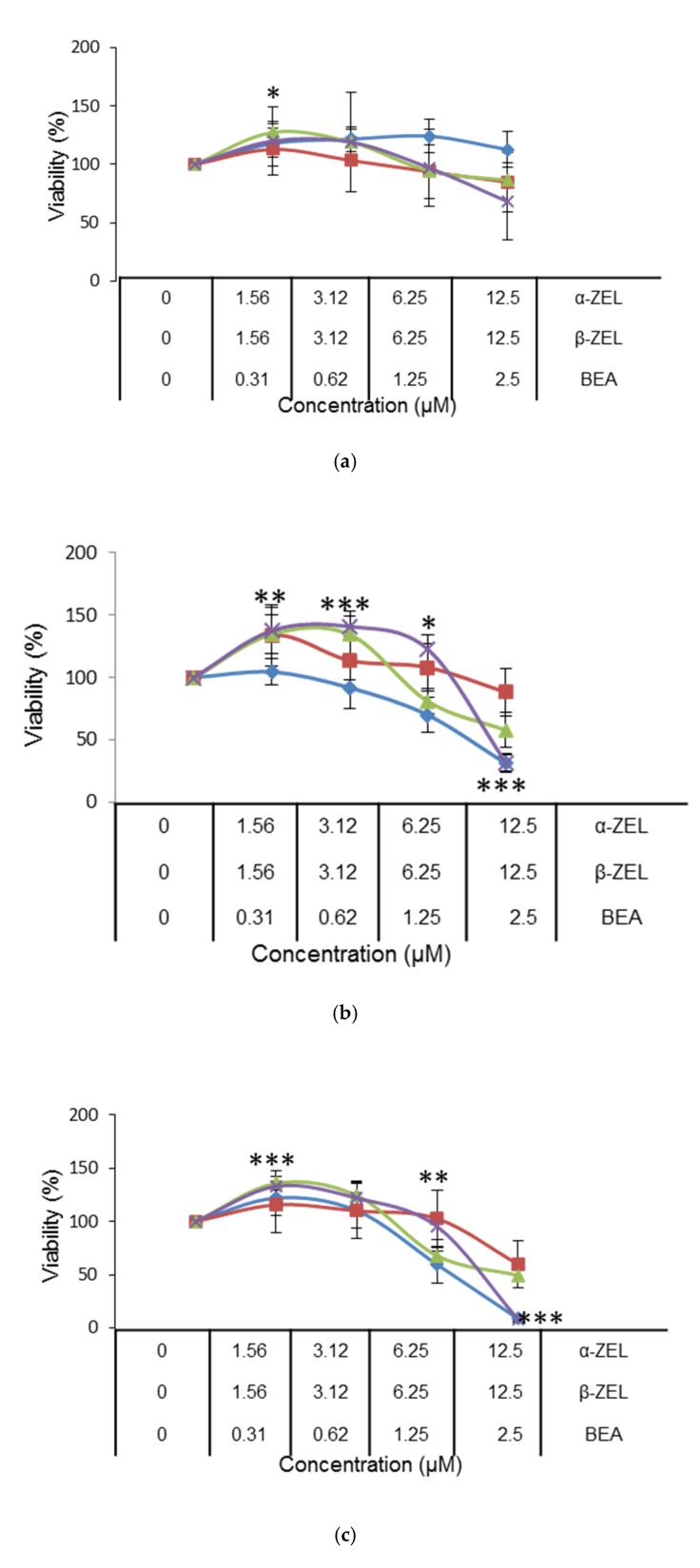
Cytotoxicity of the mycotoxin combination of α-ZEL + β-ZEL + BEA (5:5:1) at 24 h (**a**), 48 h, (**b**) and 72 h (**c**). All values are the results of three independent experiments with eight replicates and are expressed as mean ± SD; *p* ≤ 0.05 (*), *p* ≤ 0.01 (**), *p* ≤ 0.001 (***). BEA: line and square; β-ZEL: line and diamond; α-ZEL: line and triangle; Mixture: line and ×.

The cytotoxic effect of binary and tertiary combinations of α-ZEL, β-ZEL, and BEA on SH-SY5Y cells was evaluated by the MTT assays over 24, 48, and 72 h. The dose–response curves of the two- and three-mycotoxin combinations are shown in Figure 2 and Figure 3, which demonstrate higher cytotoxicity of the combinations compared with individual mycotoxin. Figure 2 shows the concentration-dependent decrease in SH-SY5Y cell viability upon combined treatment with α-ZEL + BEA (5:1) (Figure 2a), β-ZEL + BEA (5:1) (Figure 2b), α-ZEL + β-ZEL (1:1) (Figure 2c); Figure 3 shows the results for α-ZEL + β-ZEL + BEA (5:5:1).

The α-ZEL + BEA combination at the highest concentration induced a decrease in cell proliferation at 24 h of exposure (Figure 2a) of 35% with respect to the effect α-ZEL tested individually and of 37% with respect to the effect BEA. After 48 h of exposure, the decrease in cell proliferation was 67% with respect to that measured for α-ZEL and 36% with respect to that measured for BEA. After 72 h of exposure, the viability decreased 53% with respect to α-ZEL and 43% with respect to BEA. After 24 h of exposure, the β-ZEL + BEA combination (Figure 2b) decreased cell proliferation by about 55% and 29% at the highest concentration with respect to β-ZEL and BEA tested individually, respectively. After 48 h of exposure, the highest concentration of the combination reduced cell proliferation by 11% with respect to BEA tested individually. Also, at 72 h of exposure, the combination decreased cell proliferation by approximately 36% with respect to BEA individually tested. Such effect was not noticed after 48 and 72 h with respect to b-ZEL. In Figure 2c, the α-ZEL + β-ZEL combination after 24 h of exposure showed 17% of decrease in cell proliferation compared to β-ZEL individually assayed. After 48 and 72 h of exposure, the highest concentration of the combination reduced cell proliferation by 60% and 50%, respectively, compared to α-ZEL tested alone, whereas, this did not happen with respect to β-ZEL after 48 and 72 h of exposure. Figure 3 shows the dose–response curves for the tertiary combination of α-ZEL, β-ZEL, and BEA at 24, 48, and 72 h of exposure in SH-SY5Y cells. At 24 h of exposure, cell proliferation decreased by 16%, 44%, and 18% compared to cells exposed to α-ZEL, β-ZEL, and BEA alone. After 48 and 72 h of exposure, a significant reduction in cell proliferation, corresponding to 57% and 51%, was observed with respect to α-ZEL alone, and a reduction of 26% and 41% was observed with respect to BEA alone, while such effect was not observed with respect to β-ZEL alone. 

**Figure 4:**
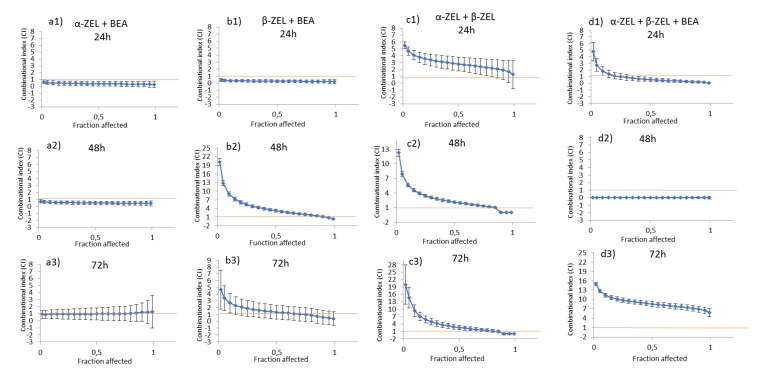
CI vs. fractional effect curve, as described by Chou and Talalay, for SH-SY5Y cells exposed to α-ZEL, β-ZEL, and BEA in binary and tertiary combinations. Each point represents the CI ± SD at a fractional effect as determined in our experiments. The line (CI = 1) indicates additivity, the area under this line indicates synergism, and the area above the line indicates antagonism. SH-SY5Y cells were exposed for 24, 48, and 72 h to α-ZEL + BEA and β-ZEL + BEA at a molar ratio of 5:1 (equimolar proportion), to α-ZEL + β-ZEL at a molar ratio of 1:1, and to α-ZEL + β-ZEL + BEA at a molar ratio of 5:5:1.

The isobologram analysis was used to determine the type of interaction between α-ZEL, β-ZEL, and BEA. The values of the parameters *Dm*, *m*, and *r* of the double and triple combinations, as well as of the mean combination index (CI) are shown in Table 2. The IC_50_, IC_75_, and IC_90_ are the doses required to inhibit proliferation at 25%, 50%, 75%, and 90%, respectively. These CI values were calculated automatically by the computer software CalcuSyn. The CI fractional effect (*fa*) curves for α-ZEL, β-ZEL, and BEA combinations in SH-SY5Y cells are shown in Figure 4. Synergism for all concentration of the α-ZEL + BEA (5:1) mixture after 24 and 48 h of exposure was demonstrated; however, after 72 h of exposure, an additive effect for the α-ZEL + BEA combination was observed (Figure 4a, Table 2). The β-ZEL + BEA (5:1) mixture showed synergism after 24 h of exposure; however, after 48 and 72 h it showed antagonism at high concentrations and moderate synergism at low concentrations (Figure 4b, Table 2). The mixture of α-ZEL + β-ZEL showed antagonism after 24 h of exposure at all concentrations assayed but at 48 and 72 h, it showed antagonism at high concentration and a moderate synergism at low concentration (Figure 4c, Table 2). The tertiary mixture, after 24 h of exposure, showed antagonism at high concentration and synergism at low concentration, while after 48 h, it showed synergism and after 72 h, antagonism at all concentrations assayed (Figure 4d, Table 2).

Cytotoxicity after 24 h of incubation decreased in this order: α-ZEL + BEA > β-ZEL + BEA > α-ZEL + β-ZEL + BEA > α-ZEL + β-ZEL. After 48 and 72 h of incubation, the ranking was α-ZEL + BEA > β-ZEL + BEA >α-ZEL + β-ZEL > α-ZEL + β-ZEL + BEA.

### 2.2. α-ZEL, β-ZEL, and BEA Present in Cell Medium after Treatment in Binary and Tertiary Combination

**Figure 5:**
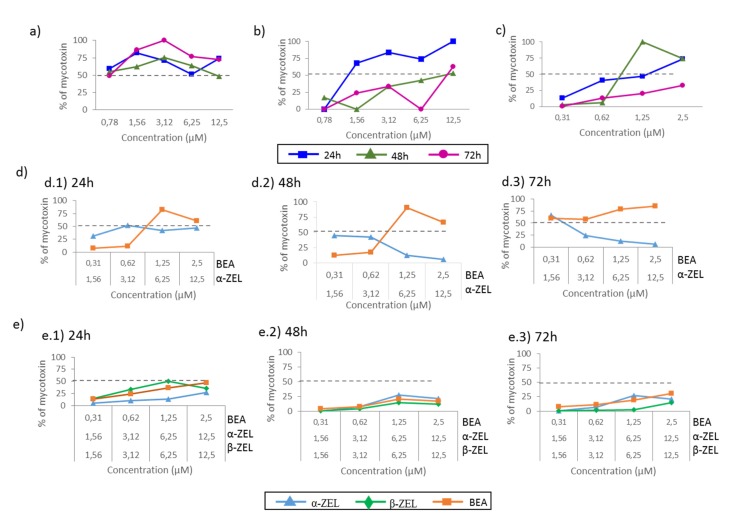
Percentage of α-ZEL, β-ZEL, and BEA remaining in the medium of SH-SY5Y cells after treatment for 24, 48, and 72 h at different concentrations individually or in combination by LC–ESI–qTOF-MS. (**a**) α-ZEL; (**b**) β-ZEL; (**c**) BEA; (**d**) α-ZEL + BEA and (**e**) α-ZEL + β-ZEL + BEA.

The medium of SH-SY5Y cells containing α-ZEL, β-ZEL, and BEA after treatments (individual and combined after 24, 48, and 72h) was collected from each well. The amount of each mycotoxin remaining in the medium was calculated as a percentage with respect to the respective amount used in the exposure assays. In this sense, we determined whether the amounts were above or below 50% of those used for treatment (Figure 5). In individual exposures, the amounts of BEA and β-ZEL in the medium were below 50% at 48 and 72 h (Figure 5b,c), while, at 24 h, their concentrations tended to be higher and >50% for both mycotoxins. For α-ZEL, the concentration in the medium was maintained above 50% at all times studied (Figure 5a). This evidenced that a lower amount of α-ZEL exerted the examined effect compared to the amount necessary for BEA and β-ZEL, as higher amounts of α-ZEL were detectable in the medium at all times and concentrations. 

In the binary combination α-ZEL + BEA (5:1), the amounts of each mycotoxin after 24 and 48 h were below 50% (Figure 5d.1,d.2), although the amount of BEA was higher than that of α-ZEL once the concentration assayed overpassed 0.62 µM for BEA and 3.12 µM for α-ZEL, revealing that the effects exerted by this mixture in neuroblastoma cells depended on both mycotoxins and were due more to α-ZEL than to BEA. This tendency at 72 h was more accentuated, as the amount of BEA in the medium was above 50% for all concentrations, while that of α-ZEL was below 50% (Figure 5d.3). 

Also, for the combination β-ZEL + BEA (5:1), the mycotoxin’s percentage remaining in the media was the same as that found for α-ZEL + BEA; however, β-ZEL was detected in higher amount than BEA in all scenarios, revealing that the effect of this mixture and was due more to BEA than to β-ZEL (Supplementary Figure S1A). On the other hand, for the binary combination of ZEN metabolites, α-ZEL + β-ZEL (1:1), the amounts of mycotoxins recovered were below 50%, and slightly superior for α-ZEL than for β-ZEL. This revealed that both mycotoxins contributed to the effect of this mixture in SH-SY5Y cell line (Supplementary Figure S1B). For the tertiary combination (α-ZEL + β-ZEL + BEA, (5:5:1)), the mycotoxins’ percentages detected were also below 50% of the administered concentration, and this percentage was higher for higher concentrations administered and lower time of exposure (Figure 5e). This revealed that high amounts of α-ZEL and β-ZEL accessed the neuroblastoma cells, and the effect was due more to β-ZEL at 48 and 72 h, according to the results in Figure 3 and Figure 5.

## 3. Discussion

Several studies have discussed the cytotoxic and an anti-proliferative effect of ZEN mycotoxin and its metabolites in various cell lines, such as Caco-2 [11], HepG2 cells [13], CHO-K1 cells [32], and SH-SY5Y [6], and hose of BEA mycotoxin in Caco [14], CHO-K1 [19], and Hep G2 cells [17]. However, there are no reports on the effect of ZEN metabolites and BEA in neuronal cells. In the present study, we proved the toxicity of ZEN metabolites (α-ZEL and β-ZEL) and BEA in human neuroblastoma SH-SY5Y cells in relation to exposure time, mycotoxin concentration, and mixture of mycotoxins.

According to the IC_50_ values of single mycotoxins, β-ZEL was the most cytotoxic mycotoxin compared to the other mycotoxins assayed individually, which is in accordance with Marin et al. (2019) [33] who studied the cytotoxicity of ZEN and its metabolites in HepG2 cells, individually and in double combinations. On the contrary, Tatay et al. (2014) [32] demonstrated that α-ZEL was the most cytotoxic among three mycotoxins tested (α-ZEL, β-ZEL, and ZEN) in CHO-K1 cells. Regarding to double combinations, it was revealed that presence of two mycotoxins increased the cytotoxic potential in SH-SY5Y cells, as shown by the lower IC_50_ values. According to Figure 2a, IC50 for α-ZEL and BEA was not reached in individual treatment however, binary combination α-ZEL + BEA (5:1) inhibited cell proliferation from up to 50 to 90% for all times studied. For the β-ZEL + BEA (5:1) binary combination, as it can be observed in Figure 2b, the IC_50_ values at 48 and 72 h were lower than that of β-ZEL. This was also observed when β-ZEL was combined with α-ZEL, for which combination (α-ZEL + β-ZEL (1:1)), the IC_50_ value was the same as that found for β-ZEL alone. This result was not achieved by Tatay et al. (2014) [31] in CHO-K1 cells, although the mycotoxin concentrations studied in binary assays in that work were two times higher than the concentrations assayed in our study. The proliferation of CHO-K1 cells treated with the α-ZEL + β-ZEL mixture at the highest concentration decreased only by 20% with respect to the values found when each mycotoxin was tested alone. In addition, in that study, the IC_50_ value was never reached for binary mixtures, whereas in our study in SH-SY5Y cells, after 48 and 72 h, the α-ZEL + β-ZEL combination inhibited cell proliferation up to 70% and 90%, respectively (Figure 2c). For the triple combination (α-ZEL + β-ZEL + BEA, (5:5:1)), cell proliferation inhibition was lower than when β-ZEL was assayed individually, and the same result was found for β-ZEL + BEA after 48 and 72 h and for α-ZEL + β-ZEL after 48 h in SH-SY5Y cells. This is in contrast with the results obtained for the tertiary combination of α-ZEL + β-ZEL + ZEN in CHO-K1 cells, as this combination was more cytotoxic than each mycotoxin tested alone [30].

As the co-occurrence of mycotoxins in food and feed is very common, some studies evaluated the toxicity and cytotoxicity of several mycotoxins, both individually and in combination, in different cell lines, using the isobologram model. In these experiments, HepG2 cells were exposed to ochratoxin A (OTA) and BEA [16], to double and triple combinations of alternariol, 3-acetyl-deoxynivalenol, and 15-acetyl-deoxynivalenol [28], and to combinations of ZEN and OTA or α-ZEL (tested also individually) [33], CHO-K1 cells in vitro were used to examine the interactions between the mycotoxins beauvericin, deoxynivalenol (DON), and T-2 toxin [26] as well as the combination of BEA, patulin, and ZEN [17], whereas Caco-2 cells were exposed to DON, ZEN, and Aflatoxin B1 [34]. It is important to understand whether the interaction between mycotoxins shows synergism, additive effects, and/or antagonism concerning cell viability. 

In SH-SY5Y cells, almost all the combinations tested reduced cell viability more than the individual mycotoxins, except the β-ZEL + BEA (5:1), α-ZEL + β-ZEL (1:1), and α-ZEL + β-ZEL + BEA (5:5:1) combinations, for which the reduction in cell viability was not significantly different from that obtained when β-ZEL was assayed individually. According to Dong et al. (2010) [5], ZEN is degraded more efficiently to α-ZEL than to β-ZEL in almost all tissues, whereas it is converted more efficiently to β-ZEL than to α-ZEL in liver and lungs. Some studies demonstrated that β-ZEL is more cytotoxic than α-ZEL [31,35,36], whereas other studies found that α-ZEL is more cytotoxic [30,35]. Hence, there is a necessity to clarify the cytotoxicity of these two mycotoxins with studies of the toxicity mechanisms involved.

The IC_50_ values obtained by the MTT assay and the amount of mycotoxin detected in the media by LC–ESI–qTOF-MS were determined and translated into percentage values as an attempt to calculate the amount of each mycotoxin involved in the cytotoxic effect and in the type of interaction effect. Hence, the percentage of mycotoxin present in the media was considered in accordance to the IC_50_ value obtained from the MTT assay (Table 1). The results showed that among the individual mycotoxins assayed, the amount of α-ZEL that remained in the culture medium was above 50% of the administered quantity at all times assayed (Figure 5a). This can be related to the effect in Figure 1a, which shows that the viability was above 100% for the doses reported in Figure 5. This can be justified by the chemical structure of this compound, which might impede its access in the cell. Our results suggest that the availability and capacity of the tested mycotoxins to get into cells were greater than those of α-ZEL, and as a consequence, the amounts of these mycotoxins detected in the media were lower than that of α-ZEL. To notice that the higher the amount of mycotoxin in the medium (at 24 h), the higher the cell viability, which might be related to the lower amount of mycotoxin affecting the live cells. On the contrary, BEA seemed to have easier access the cells, as its percentage in the medium was generally below 50%, but cell viability was maintained above 50% for the doses assayed, indicating the lower potential toxicity of BEA in SH-SY5Y cells compared to ZEN metabolites. In fact, among all three mycotoxins tested, BEA reached the IC_50_ values after long exposures times (72 h) (Table 1 and Figure 1c), highlighting again the mild toxic effect of BEA in SHY-SY5Y cells compared to ZEN metabolites.

According to this and when analyzing combinations, the amounts of ZEN metabolites found in the medium were in most cases below BEA’s amounts, indicating easier access of these compounds in SH-SY5Y compared to BEA. In detail, for the α-ZEL + BEA combination (Figure 2a), it can be observed that the lower the amount of α-ZEL in the medium over time (Figure 5d), the lower the viability of SH-SY5Y cells, in particular at 72h. For triple mixtures, the cytotoxic effect was weaker at all times and for all mixtures compared with that of binary combinations; however, the amounts of each mycotoxin detected were all below 50%, and the cytotoxic effect seemed to be bearable for SH-SY5Y cells for doses administered in the first and second mixture but not for those of the third mixture (6.25 + 6.26 + 1.25) µM (α-ZEL + β-ZEL + BEA, 5:5:1), specifically at 48 and 72 h. We suggest that cytotoxicity is due to the stimulation of different biochemical mechanisms that, after a certain level of stimulation, cannot be controlled and cause cell death. Therefore, it is necessary to study in detail the mechanisms of action implicated in the cytotoxic effects that occur when several mycotoxins are present in the same food or diet.

## 4. Conclusions

In conclusion, the treatment with β-ZEL alone presented the highest cytotoxicological potency compared to treatments with the other mycotoxins assayed (α-ZEL and BEA). The main type of interaction detected between mycotoxins for all combinations assayed was synergism. The potential interaction effects between combinations in this study are difficult to explain since α-ZEL + BEA for binary and α-ZEL + β-ZEL + BEA for tertiary combination were found more in favor of synergic effect respect to CI value, compared with other combinations, which could be related to the concentration range studied, ratio in each mixture, exposure time assayed and cell line studied. Moreover, among all mycotoxins assayed, α-ZEL appeared to remain in the culture medium and was less able to get into SH-SY5Y cells compared to BEA and β-ZEL. In combinations, such effect was observed for BEA reaching the highest in α-ZEL + BEA.

## 5. Materials and Methods 

### 5.1. Reagents

**Figure 6:**
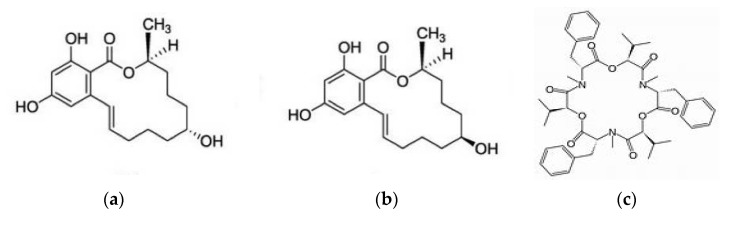
Chemical structures of the mycotoxins (**a**) α-ZEL, (**b**) β-ZEL, and (**c**) BEA.

The reagent-grade chemicals and cell culture components used, Dulbecco’s Modified Eagle’s Medium- F12 (DMEM/F-12), fetal bovine serum (FBS), and phosphate-buffered saline (PBS) were supplied by Thermofisher, Gibco ™ (Paisley, UK). Methanol (MeOH, HPLC LS/MS grade), was obtained from VWR International (Fontenay-sous-Bois, France). Dimethyl sulfoxide was obtained from Fisher Scientific Co, Fisher BioReagnts ™ (Geel, Belgium). The compound (3-(4,5-dimethylthiazol-2-yl)-2,5-diphenyltetrazolium bromide) (MTT) for the MTT assay, penicillin, streptomycin, and Trypsin–EDTA were purchased from SigmaAldrich (St. Louis, MO, USA). Deionized water (<18, MΩcm resistivity) was obtained in the laboratory using a Milli-QSP^®^ Reagent Water System (Millipore, Beadford, MA, USA). Standard BEA (MW: 783.95 g/mol), α-ZEL, and β-ZEL (MW: 320.38 g/mol) were purchased from SigmaAldrich (St. Louis Mo. USA) (Figure 6). Stock solutions of mycotoxins were prepared in MeOH (α-ZEL and β-ZEL) and DMSO (BEA) and maintained at −20 °C in the dark. The final concentration of either methanol or DMSO in the medium was ≤1% (v/v) as previously established. All other reagents were of standard laboratory grade.

### 5.2. Cell Culture 

The human neuroblastoma cell line SH-SY5Y was obtained from the American Type Culture Collection (ATCC, Manassas, VA, USA) and cultured in Dulbecco’s Modified Eagle’s Medium/F12 (DMEM/F-12), supplemented with 10% FBS, 100 U/mL penicillin, and 100 mg/mL streptomycin. The cells were sub-cultivated after trypsinization once or twice a week and suspended in complete medium in a 1:3 split ratio. The cells were maintained as monolayers in 150 cm^2^ cell culture flasks with filter screw caps (TPP, Trasadingen, Switzerland). Cell cultures were incubated at 37 °C, 5% CO_2_ atmosphere.

### 5.3. Mycotoxin Exposure

Concentration of the mycotoxins and exposure time are two factors that were considered to in this study. The cells were exposed to α-ZEL, β-ZEL, and BEA mycotoxins individually for 24, 48, and 72 h at a concentration in the ranges of 0.39 to 100 μM for α-ZEL and β-ZEL and 0.009 to 25 μM for BEA, all with 1:2 dilution (Table 3). Also, the mycotoxins were assayed in combination in the following mixtures: α-ZEL + BEA, β-ZEL + BEA, α-ZEL + β-ZEL, and α-ZEL + β-ZEL + BEA at three exposure times 24, 48, and 72 h. The concentrations ranged from 1.87 to 25 μM for the binary combinations were studied and from 3.43 to 27.5 μM for the tertiary combination, including four dilutions of each mycotoxin: BEA (0.31, 0.62, 1.25, and 2.5 μM), α-ZEL and β-ZEL (1.56, 3.12, 6.25 and 12.5 μM) (Table 3). The dilution ratios of the concentrations for the binary combinations were 5:1 for α-ZEL + BEA and β-ZEL + BEA, 1:1 for α-ZEL + β-ZEL, and 5:5:1 for the tertiary combination (β-ZEL + α-ZEL + BEA) (Table 3). 

### 5.4. MTT Assay

Cytotoxicity was examined by the MTT assay, performed as described by Ruiz et al. (2006) [37], with few modifications. The assay consists in measuring the viability of cells by determining the reduction of the yellow soluble tetrazolium salt only in cells that are metabolically active via a mitochondrial reaction to an insoluble purple formazan crystal. Cells were seeded in 96-well culture plates at 2 × 96 cells/well and allowed to adhere for 18–24 h before mycotoxin additions. Serial dilutions of α-ZEL, β-ZEL, and BEA at 1:2 dilutions were prepared with supplemented medium and added to the respective plates (Table 3). Culture medium without mycotoxins and with 1% MeOH or DMSO was used as a control. After treatment, the medium was removed, and each well received 200 μL of fresh medium containing 50 μL of MTT solution (5 mg/mL; MTT powder dissolved in phosphate-buffered saline). After an incubation time of 4 h at 37 °C in the darkness, the MTT-containing medium was removed, and 200 μL of DMSO and 25 μL of Sorensen’s solution were added to each well before reading the optical density at 620 nm with the ELISA plate reader Multiskan EX (Thermo Scientific, MA, USA). Each mycotoxin combination plus a control were tested in three independent experiments. Mean inhibition concentration (IC_50_) values were calculated from full dose–response curves.

### 5.5. Experimental Design and Combination Index

The isobologram analysis (Chou–Talalay model) was used to determine the type of interaction (synergism, additive effect, and antagonism) that occurred when the mycotoxins studied were in combination. This model allows characterizing the interactions induced by combinations of mycotoxins in different cell lines and with different mycotoxins but it does not allow the elucidation of the mechanisms by which these types of interaction are produced. The median effect/combination index (CI) isobologram equation by Chou (2006) [31] and Chou and Talalay (1984) [30] permitted analyzing drug combination effects. The isobologram analysis involves plotting the dose–effect curves for each compound and its combinations in multiple diluted concentrations. Parameters such as *Dm* (median effect dose), *fa* (fraction affected by concentration), and *m* (coefficient signifying the shape of the dose–effect relationship) are relevant in the equation [30]. Therefore, the method considers both potency (*Dm*) and shape (*m*) parameters. 

Chou and Talalay (1984) [30] introduced the term combination index (CI). CI values <1, =1, and >1 indicate synergism, additive effects, and antagonism of the combination, respectively. CalcuSyn software version 2.1. (Biosoft, Cambridge, UK, 1996–2007) was used to study the types of interactions assessed by the isobologram analysis. The IC_25_, IC_50_, IC_75_, and IC_90_ are the doses required to produce toxicity at 25%, 50%, 75%, and 90%, respectively.

### 5.6. Extraction of α-ZEL, β-ZEL, and BEA from the Culture Media

To determine the intracellular accumulation of the mycotoxins studied, an extraction procedure of the culture media was carried out following the method described by Juan-García et al. (2015 and 2016) [27,28], with several modifications. Briefly, 0.8 mL of culture medium was collected and transferred into a polypropylene tube, 1.5 mL of ethyl acetate was added, and the mixture was shaken for 2 min with an Ultra-Turrax Ika T18 basic (Staufen, Germany). Afterwards, the mixture as sonicated in an ultrasound cleaning bath (VWR, USC1700TH) for 10 min. Finally, the mixture was centrifuged at ~5600× *g* for 5 min at 22 °C (Centrifuge 5810R, Eppendorf, Germany). The supernatant phase was collected. The liquid–liquid extraction process was repeated three times. Finally, the total volume obtained (approx. 4.5 mL) was evaporated to dryness at 45 °C in an N2 stream with a TurboVap-LV (Zymark, Allschwil, Switzerland) and then re-dissolved in 0.25 mL of a mixture of methanol and water (70:30, v/v) by vortexing vigorously (15 s), before being transferred into a vial for LC–ESI–qTOF-MS injection. 

### 5.7. Determination of BEA, β-ZEL, and α-ZEL by LC–ESI–qTOF-MS

The analysis was performed using an LC–ESI–qTOF-MS system, consisting of an LC Agilent 1200-LC system (Agilent Technologies, Palo Alto, CA, USA) equipped with a vacuum degasser, an autosampler, and a binary pump. The columns were a Gemini NX-C18 column (150 × 2 mm, i.d. 3 μm, Phenomenex, Torrance, California) and a guard column C18 (4 × 2 mm, i.d. 3 μM).

Mobile phases consisted of milli-Q water with 0.1% of formic acid as solvent system A and acetonitrile and 0.1% of formic acid as solvent system B, with the following gradient elution: 3 min, 70% B; in 2 min 70–80% B; in 1 min get 90% of B, maintained 4 min; 90–100% B 4 min and maintained 2 min; in 2 min decrease to 50% B; in 2 min 90% B, maintained 2 min. The flow rate used was 0.250 mL min^−1^, and the total run time was 22 min. The sample volume injected was 20 μL.

MS analysis was carried out using a 6540 Agilent Ultra- High-Definition Accurate-Mass q-TOF-MS, equipped with an Agilent Dual Jet Stream electrospray ionization (Dual AJS ESI) interface in negative and positive ionization modes. Operation conditions were as follows: sheath gas temperature 350 °C at a flow rate of 8 L/min, capillary voltage 3500 V, nebulizer pressure 45 psig, drying gas 10 L/min, gas temperature 300 °C, skimmer voltage 65 V, octopole RF peak 750 V, and fragmentor voltage 130 V. Analyses were performed using AutoMS/MS mode with fixed collision energy (10, 20 and 30) and in mass range of 50–1700 *m*/*z*. Acquisition rate was 3 spectra/second. Acquisition data were processed with Agilent MassHunter Workstation software.

### 5.8. Statistical Analysis

Statistical analysis of data was carried out using IBM SPSS Statistic version 23.0 (SPSS, Chicago, Il, USA) statistical software package. Data are expressed as mean ± SD of three independent experiments. The statistical analysis of the results was performed by student’s T-test for paired samples. Difference between groups were analyzed statistically with ANOVA followed by the Tukey HDS post-hoc test for multiple comparisons. The level of *p* ≤ 0.05 was considered statistically significant.

## Figures and Tables

**Table 1:** Medium inhibitory concentration (IC_50_ ± SD) of α-zearalenol (α-ZEL), β-zearalenol (β-ZEL), and beauvericin (BEA) for SH-SY5Y cells after 24, 48, and 72 h of exposure, determined by the MTT assay. Three independent experiments were performed with eight replicates each.

Mycotoxin	IC_50_ (µM) ± SD		
	24 h	48 h	72 h
α-ZEL	n.a	20.8 ± 0.5	14.0 ± 1.8
β-ZEL	94.3 ± 2.0	9.1 ± 1.8	7.5 ± 1.2
BEA	n.a	n.a	2.5 ± 0.2

n.a: not available.

**Table 2:** The parameters *Dm*, *m*, and *r* are the antilog of x-intercept, the slope, and the linear correlation of the median-effect plot, which means the shape of the dose–effect curve, the potency (IC_50_), and the conformity of the data to the mass action law, respectively [30,31]. *Dm* and *m* values are used for calculating the combination index (CI) value (CI < 1, =1, and >1 indicate synergism (Syn), additive (Add) effect, and antagonism (Ant), respectively. IC_50,_ IC_75_, and IC_90_ are the doses required to inhibit proliferation at 50%, 75%, and 90%, respectively. CalcuSyn software automatically provided theses values.

Mycotoxin	Time (h)	Dm (µM)	m	r	IC Values					
					IC_50_		IC_75_		CI_90_	
α-ZEL	24	66.10	1.36	0.9679						
	48	31.59	1.82	0.9726						
	72	15.24	2.02	0.9873						
										
β-ZEL	24	171.33	1.28	0.9709						
	48	12.46	1.26	0.9715						
	72	11.65	2.28	0.9464						
										
BEA	24	21.65	0.98	0.9763						
	48	3.68	1.24	0.9945						
	72	2.59	1.40	0.9805						
										
α-ZEL+BEA	24	3.05	1.36	0.9736	0.37 ± 0.33	Syn	0.34 ± 0.35	Syn	0.31 ± 0.38	Syn
	48	1.16	1.56	0.9933	0.50 ± 0.24	Syn	0.47 ± 0.26	Syn	0.44 ± 0.29	Syn
	72	1.34	1.54	0.94708	0.96 ± 0.86	Add	1.00 ± 0.51	Add	1.20 ± 1.30	Ant
										
β-ZEL+BEA	24	3.78	1.20	0.9698	0.29 ± 0.19	Syn	0.26 ± 0.21	Syn	0.24 ± 0.24	Syn
	48	4.81	3.04	0.7744	3.24 ± 0.42	Ant	1.94 ± 0.32	Ant	1.00 ± 0.14	Add
	72	1.89	3.14	0.7585	1.35 ± 0.51	Ant	1.00 ± 0.12	Add	0.60 ± 0.52	Syn
										
α-ZEL+β-ZEL	24	133.46	1.73	0.7782	2.80 ± 1.01	Ant	2.32 ± 0.51	Ant	1.92 ± 0.62	Ant
	48	19.12	3.40	0.7782	2.14 ± 0.23	Ant	1.35 ± 0.18	Ant	0.30 ± 0.14	Syn
	72	7.89	5.01	0.9409	2.60 ± 0.90	Ant	1.42 ± 0.63	Ant	0.45 ± 0.42	Syn
										
α-ZEL+β-ZEL+BEA	24	3.74	3.14	0.9478	0.57 ± 0.30	Syn	0.32 ± 0.20	Syn	0.19 ± 0.14	Syn
	48	0.01	0.43	0.7465	0.23 ± 0.06	Syn	0.15 ± 0.07	Syn	0.18 ± 0.10	Syn
	72	7.47	2.30	0.8966	8.54 ± 0.77	Ant	7.60 ± 0.85	Ant	6.88 ± 0.95	Ant

**Table 3:** Concentration range (μM) of mycotoxins studied individually and in combinations. The dilution ratios were 5:1 for the combinations α-ZEL + BEA and β-ZEL + BEA, 1:1 for α-ZEL + β-ZEL, and 5:5:1 for α-ZEL + β-ZEL + BEA.

Combination Tested	Concentration Range (μM)
α-ZEL	(0.39–00)
β-ZEL	(0.39–100)
BEA	(0.009–25)
α-ZEL + BEA	(1.56–2.5) + (0.31–2.5)
β-ZEL + BEA	(1.56–2.5) + (0.31–2.5)
α-ZEL + β-ZEL	(1.56–12.5) + (1.56–12.5)
α-ZEL + β-ZEL + BEA	(1.56–12.5) + (1.56–12.5) + (0.31–2.5)

## Supplementary Materials

The following are available online at https://www.mdpi.com/2072-6651/12/4/212/s1, Figure S1. Percentage of α-ZEL, β-ZEL, and BEA remaining in the medium of SH-SY5Y cells after treatment during 24, 48, and 72 h at different concentrations and combinations by LC–ESI–qTOF-MS. (**A**) β-ZEL+ BEA and (**B**) α-ZEL + β-ZEL.
