# Supplementary material for: Individual and Combined Effect of Zearalenone Derivates and Beauvericin Mycotoxins on SH-SY5Y Cells
Source: Toxins (Basel). 2020 Mar 27;12(4):212. doi: 10.3390/toxins12040212 (PMC7232440; doi:10.3390/toxins12040212)
Supplement: Supplementary file 1 [file toxins-12-00212-s001.pdf]

Supplementary Materials: Individual and Combined Effect of Zearalenone Derivates and Beauvericin Mycotoxins on SH-SY5Y Cells

Fojan Agahi, Guillermina Font, Cristina Juan and Ana Juan-García

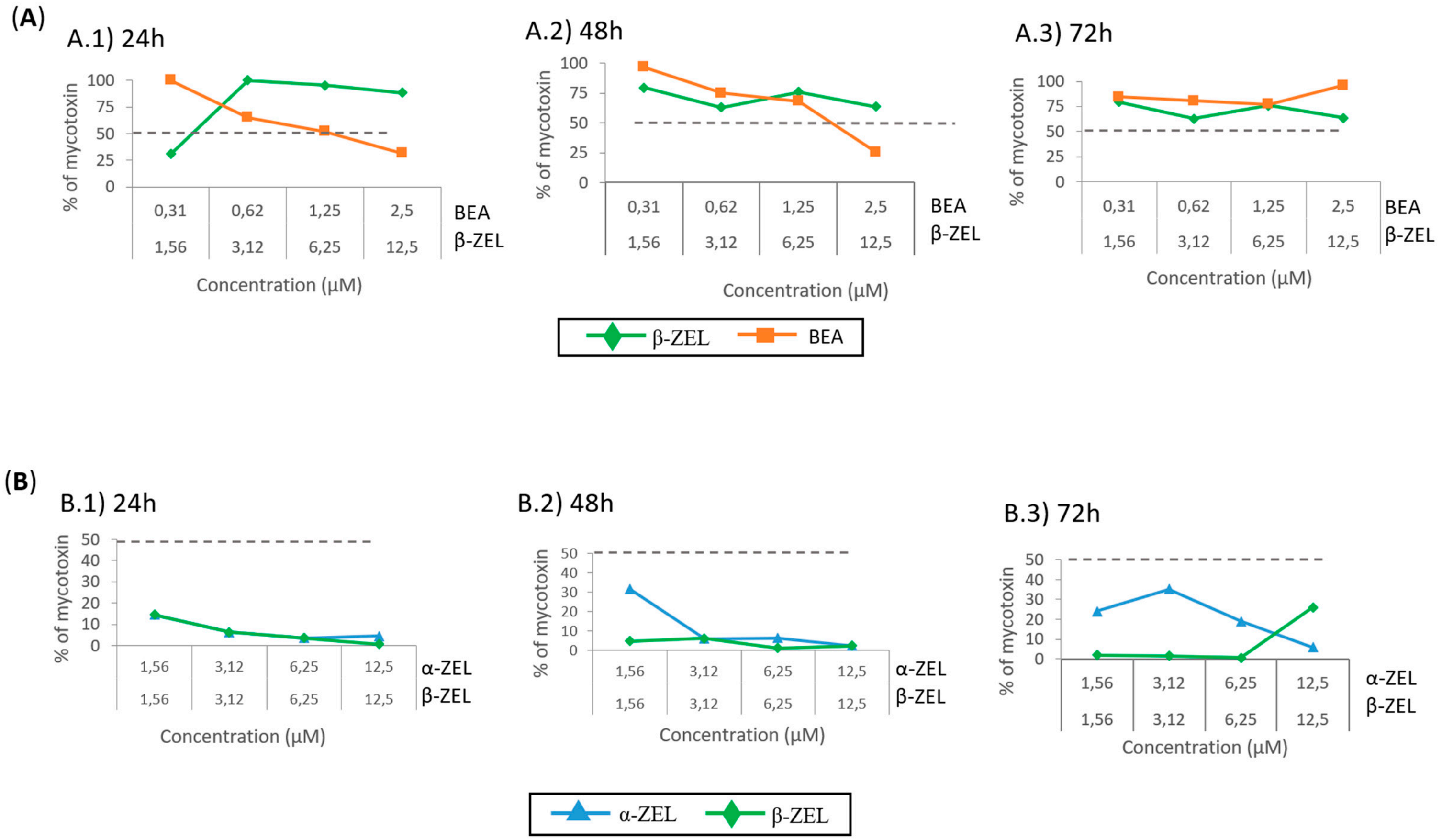

Figure S1. Percentage of α-ZEL, β-ZEL, and BEA remaining in the medium of SH-SY5Y cells after treatment during 24, 48, and 72 h at different concentrations and combinations by LC–ESI–qTOF-MS. (**A**) β-ZEL+ BEA and (**B**) α-ZEL + β-ZEL.
